# The World Health Report 2012 That Wasn't

**DOI:** 10.1371/journal.pmed.1001317

**Published:** 2012-09-25

**Authors:** The PLOS Medicine Editors

## Abstract

The *PLOS Medicine* editors comment on the history of the World Health Organization's latest World Health Report, originally planned for publication in 2012, and the outcomes of the journal's collaboration with WHO on the intended theme of "no health without research".

A year and a half ago, *PLOS Medicine* announced a collaboration with the World Health Organization (WHO), inviting submission of articles to *PLOS Medicine* on the theme of “no health without research” [1]. That call for papers was intended to culminate in an open-access collection of original research and commentary articles to coincide with the launch in 2012 of a World Health Report on the same topic. The collection was to focus on eight key areas (detailed in [1]) relating to how countries can strengthen their health research systems, to better inform healthcare delivery and policymaking. The importance of a strategic, evidence-informed approach, particularly for low- and middle-income countries, is highlighted in a statement made at the 2008 Global Ministerial Forum on Research for Health, in Bamako, Mali, that “Countries don't need a national airline, but they do need a national health research strategy” [2].

The collection (available at http://www.ploscollections.org/whr2012) has been continually updated throughout 2011 and 2012, and includes articles published across the PLOS journals. It includes a wealth of studies and commentary that, for example, reflect countries' experiences with establishing and maintaining robust research systems such as developing evidence-based priority setting for maternal, neonatal, and child health in Africa [3]; the creation of regional vaccine research networks in Asia [4]; and the evaluation of research capacity strengthening programmes in low- and middle-income countries [5]. The collection also includes four commissioned pieces from leading scholars in the area that contextualize and critically reflect on the intended theme of the 2012 World Health Report [2],[6],[7],[8].

In light of the interest in the collection, it is disappointing to learn now that the 2012 World Health Report will not exist, at least as originally envisaged. Communications to WHO staff on behalf of the Director-General Margaret Chan reveal that the report has been delayed until 2013. The original webpage describing the intended report has been removed and replaced by a new page that notes the report's focus will now be oriented towards “the contributions of research to universal health coverage” [9]. The reasons for these delays and for the changes in scope of WHO's flagship publication, are unclear. Previous World Health Reports, for example the 2000 edition on “Health Systems: Improving Performance,” described as “an act of remarkable courage” [10], have represented bold political statements. Most notably, the 2000 Report, which ranked nations' health systems performance (to the delight or ire of many countries) has subsequently been described as leaving a clear legacy—for example, in stimulating critical research and filling data gaps on the performance of health systems [10]. At this stage the scope of the forthcoming Report is still vague, and linkages to the 2010 Report on Health Systems Financing are not yet apparent based on information in the public domain [9]. However, we look forward to seeing how the forthcoming Report develops, and hope that this publication makes a similarly bold and influential contribution as previous Reports have done.

We are proud of the papers that we have published as part of the current collection. PLOS would like to thank the authors who have responded enthusiastically to our call for papers and enabled us to publish such a diverse and incisive range of research and commentary reflecting the original theme of our collaboration with WHO [1]. While the 2012 World Health Report will not appear as previously envisioned, the WHO/PLOS Collection on “No Health Without Research,” now closed to new submissions, remains an important resource for investigators, policy makers, and other readers, reflecting the original intentions of both WHO and PLOS.
